# Relationships Between Regional Left Ventricular Myocardial Strain and Tissue Characteristics in Hypertrophic Cardiomyopathy

**DOI:** 10.3390/jcdd13090430

**Published:** 2026-09-02

**Authors:** Gabriela S. Galvao, Badr Bannan, Laura Jimenez-Juan, Huda S. Ismail, Faisal Alabdulkarim, Matias F. Callejas, Yin Ge, Djeven P. Deva, Andrew T. Yan

**Affiliations:** 1 Department of Medical Imaging, University of Toronto, Toronto, ON M5S 1A1, Canada; gabisg@gmail.com (G.S.G.); bannan_22@hotmail.com (B.B.); laura.jimenez-juan@unityhealth.to (L.J.-J.); dr.huda.si@gmail.com (H.S.I.); fmalabdulkarim@gmail.com (F.A.); matiasfelipe.callejascanepa@unityhealth.to (M.F.C.); 2 Department of Medical Imaging, Hospital Moinhos de Vento, Porto Alegre 90035-001, Brazil; 3 Keenan Research Centre, Li Ka Shing Knowledge Institute, St. Michael’s Hospital, Toronto, ON M5B 1W8, Canada; 4 Department of Medical Imaging, St. Michael’s Hospital, Toronto, ON M5B 1W8, Canada; 5 Department of Medicine, University of Toronto, Toronto, ON M5S 1A1, Canada; yin.ge@unityhealth.to; 6 Terrence Donnelly Heart Centre, Division of Cardiology, St. Michael’s Hospital, Toronto, ON M5B 1W8, Canada

**Keywords:** cardiovascular magnetic resonance, hypertrophic cardiomyopathy, myocardial strain, fibrosis, T1 mapping, extracellular volume fraction

## Abstract

Both fibrosis and hypertrophy contribute to abnormal myocardial mechanics in hypertrophic cardiomyopathy (HCM). We sought to assess the relationships between regional structural and functional parameters in HCM by cardiac magnetic resonance (CMR). This was a retrospective single-center study of HCM patients and age-matched controls with either hypertensive heart disease (HHD) or normal CMRs. CMR feature tracking was performed to assess global and segmental 2D-radial, circumferential, and longitudinal LV strain, while native and post-contrast T1 parametric mapping analysis was performed to assess the global and regional T1 values and ECV fraction. Of 100 patients (age 56 ± 15 years; 66% male), 62 were in the HCM group and 38 in the control group (19 healthy individuals and 19 with HHD). Compared to the control group, global circumferential strain (−16.1 ± 5.9% vs. −20.8 ± 3.6%, *p* < 0.001) and radial strain (45.2 ± 14.1% vs. 32.1 ± 14.0%, *p* < 0.001) were worse in the HCM group. Among the HCM patients, there were significant correlations between segmental native T1 values at the most hypertrophied segment and global longitudinal strain (r = 0.27, *p* = 0.031), global circumferential strain (r = 0.34, *p* = 0.006), global radial strain (r = −0.29, *p* = 0.024), and left ventricular ejection fraction (LVEF) (r = −0.31, *p* = 0.014). In HCM, higher myocardial T1 at the most hypertrophied segment correlated with worse global LV myocardial strain and LVEF by CMR.

## 1. Introduction

Myocardial deformation imaging has been shown to detect early contractile dysfunction in various cardiovascular diseases [1,2,3]. Cardiovascular magnetic resonance (CMR) is the reference standard for the evaluation of ventricular morphology and function. CMR feature tracking (CMR-FT) is a contrast-free quantitative method using cine images in routinely acquired CMR scanning to quantify left ventricular myocardial deformation or strain in longitudinal, radial and circumferential orientations. CMR-FT has recently been shown to have diagnostic and prognostic value beyond left ventricular ejection fraction (LVEF) in patients with coronary artery disease, dilated cardiomyopathy (DCM) and myocarditis [4,5,6,7,8,9]. In hypertrophic cardiomyopathy (HCM), myocardial hypertrophy with increased wall stress results in relative subendocardial ischemia and fibrosis, leading to worse (i.e., less negative) global longitudinal strain (GLS) [10]. Myocardial fibrosis is increasingly recognized as an important component of adverse remodeling in HCM and may contribute to impaired myocardial mechanics. Accordingly, non-invasive techniques that permit tissue characterization may provide important insight into the structural substrate underlying functional impairment in this disease.

Myocardial T1 mapping has emerged as a non-invasive quantitative method for detecting and characterizing myocardial tissue abnormalities. Native T1 mapping, obtained without contrast administration, is sensitive to a range of myocardial changes, including fibrosis and edema. Prior studies showed that native (non-contrast) T1 mapping can differentiate myocardial abnormality of HCM from healthy myocardium [11]. Extracellular volume (ECV) fraction, calculated using pre- and post-T1 maps and the patient’s hematocrit, provides a non-invasive assessment of the expansion of the extracellular volume and has been validated by histologic studies to correlate with diffuse interstitial fibrosis [12,13]. Elevated ECV is associated with adverse cardiac events in patients with heart failure, DCM, and coronary artery disease [14,15,16,17,18]. Elevated ECV and native T1 can also distinguish healthy individuals from patients with HCM [19], and ECV can independently predict major adverse cardiac events in HCM patients [20].

Beyond strain, myocardial work (MW) indices have emerged as physiologically richer metrics of LV mechanics. Trimarchi et al. have provided a comprehensive framework for MW methodology in echocardiography, demonstrating its utility across a spectrum of cardiomyopathies [21]. Notably, de Gregorio et al. demonstrated that MW indices can differentiate nonobstructive HCM from transthyretin cardiac amyloidosis, a clinically critical differential diagnosis [22]. These advances underscore the importance of comprehensive multimodality structural and functional characterization in HCM.

Although a myriad of important structural and functional changes occur in HCM, these relationships have not been well studied. Comprehensive assessment of both tissue characterization using T1 and ECV and myocardial function using strain may advance our understanding of the pathophysiology of HCM [20]. Therefore, the purpose of this study was to assess the relationship between the left ventricular myocardial strain and myocardial fibrosis in HCM patients.

## 2. Materials and Methods

### 2.1. Study Design

The institutional Research Ethics Board approved this retrospective study and waived the requirement to obtain informed consent. This was a retrospective, cross-sectional study of consecutive patients who underwent CMR for suspected HCM between January 2018 and April 2021. Exclusion criteria were age < 18 years, severe respiratory motion on CMR, image degradation due to arrhythmia, suboptimal T1 mapping image quality, no post-contrast T1 mapping, cases performed using 3 Tesla scanner, diagnosis of Anderson–Fabry disease, amyloidosis, iron-deposition condition (e.g., hemochromatosis), known myocardial infarction, evidence of myocarditis or pericarditis, and athletic activity with sufficient duration, intensity and frequency to explain abnormal left ventricular wall thickness (LVWT).

### 2.2. Cardiac MR Image Acquisition

CMRs were performed on a Philips 1.5T Ingenia MRI scanner (Philips Healthcare, Best, The Netherlands). Breath-hold, retrospectively electrocardiogram (ECG)-gated cine balanced steady-state free precession (bSSFP) images were obtained in long-axis views, and as a short-axis stack covering the entire LV (8 mm slices with 2 mm gaps), through 25 phases per cardiac cycle. A dose of 0.2 mmol/kg of gadolinium-based contrast agent (GBCA) was administered, followed by a saline flush. Breath-hold Modified Look-Locker Imaging (MOLLI) sequence was performed for native and post-contrast myocardial T1 parametric mapping with 3 slices in the short-axis plane at the basal, mid, and apical LV. Late gadolinium enhancement (LGE) imaging was performed in the LV long-axis views (2-, 3-, and 4-chamber) and short-axis stack 12 min after intravenous injection of the GBCA.

### 2.3. HCM Definition and Criteria

HCM diagnostic criteria included LVWT ≥ 15 mm at end-diastole anywhere in the left ventricle or septal to lateral wall thickness ratio higher than 1.3 in a non-dilated LV in the absence of loading conditions sufficient to cause the observed abnormality [23,24]. If a positive genetic test or family history of HCM were present, limited hypertrophy (13–14 mm) was diagnostic [24]. HCM was classified according to its morphology into localized basal septal hypertrophy, reverse curvature septal hypertrophy, apical HCM, concentric HCM, and mid-cavity obstruction with apical aneurysm [25].

The control group was composed of subjects spanning a clinically relevant spectrum of LVWT and mass, from individuals with normal cardiac dimensions to those with HHD. This spectrum reflects the real-world differential diagnostic context in which HCM is evaluated, where the primary clinical question is whether increased LVWT exceeds what can be explained by loading conditions, such as hypertension. Subjects with HHD (*n* = 19) were defined as having increased LVWT (>12 mm) in the presence of arterial hypertension [26] and absence of LV cavity dilatation, severe chronic kidney disease, or other cardiac disease associated with a similar magnitude of hypertrophy. All remaining control subjects (*n* = 19) had normal cardiac dimensions/volumes, normal cardiac function, and absence of LGE or a history of cardiac disease. Together, the continuous range of blood pressure, LVWT, and LV mass in these 2 subgroups constitutes the control group, for comparison with the HCM group where hypertrophy was caused by mutations rather than loading conditions.

The 16-segment model was used to divide the LV for segmental T1, ECV, strain, and wall motion analysis.

### 2.4. Cardiac MR Image Analysis

All image analysis was performed using commercially available software (CVi42 version 5, Circle Cardiovascular Imaging Inc., Calgary, AB, Canada) by a single experienced CMR reader (BB with 2 years of clinical CMR experience). LV short-axis stack steady-state free precession (SSFP) cine images were used to calculate LV volumes and LV myocardial mass by manually drawing the epicardial and endocardial contours at end-diastole and end-systole. Two-, 3-, and 4-chamber, and short-axis images were uploaded into the software for CMR-FT analysis of 2D-longitudinal, radial, and circumferential LV strain. CMR-FT analysis was performed by manually drawing the endocardial and epicardial contours at end-diastole (reference phase) using either long-axis or short-axis cine images. CVi42 automatically propagated the contours throughout the cardiac cycle, which were then manually adjusted if needed. Maximum end-diastolic wall thickness was measured in the short-axis, and the LV segment with the greatest wall thickness was selected for dedicated regional analysis.

For the native myocardial T1 mapping and ECV analysis, endocardial and epicardial contours were manually delineated at end-diastole in the basal, mid, and apical slices. The blood pool was selected by carefully avoiding papillary muscles and myocardial trabeculation. T1 values were recorded as per the standard American Heart Association (AHA) 16-segment model [27]. Regional T1 values were obtained by averaging basal, mid, and apical segmental values. Global T1 value was derived as the average of all 16 myocardial segments. ECV was calculated according to the following formula [28]: ECV = (1 − hematocrit) × ([1/T1myo post − 1/T1myo pre]/[1/T1blood post − 1/T1blood pre]). Specific analysis was also performed in the most hypertrophied segment by contouring a region of interest in that segment on pre- and post-contrast T1 maps to obtain corresponding segmental native T1 and ECV values. Local normal ranges for these sequences were 942–1074 ms for native T1 and 20–31% for ECV [29].

LV short-axis stack of LGE images was analyzed using the same software. The endocardial and epicardial contours were manually traced in each slice of the short-axis stack images. The LGE was visually detected [30].

### 2.5. Statistical Analysis

Categorical values are expressed as frequencies and percentages and compared using the Chi-square test. Continuous variables are presented as mean ± standard deviation (SD) and compared using a *t*-test for normally distributed data and Mann–Whitney U test for non-normally distributed data. Relationships between structural parameters (native T1, ECV, maximum end-diastolic wall thickness, and indexed LV mass [LVMI]) versus functional parameters (global longitudinal strain [GLS], global radial strain [GRS], global circumferential strain [GCS], LVEF) were evaluated by Spearman’s correlation coefficients (r). To further examine the relationship between structure and function (GCS and GLS) in HCM, we performed multivariable analysis adjusting for sex and tertiles of native T1 and end-diastolic wall thickness in the most hypertrophied segment. A two-sided *p*-value less than 0.05 was considered statistically significant. Statistical analysis was performed using SPSS software (IBM SPSS Statistics for Windows, Version 26.0. Armonk, NY, USA: IBM Corp.).

No formal a priori sample size calculation was performed, as no prior CMR studies have estimated correlation coefficients between tissue characterization and functional parameters in HCM. The study population therefore comprised all consecutive eligible HCM patients and controls during the study period.

## 3. Results

### 3.1. Baseline Characteristics and CMR Parameters

**Figure 1:**
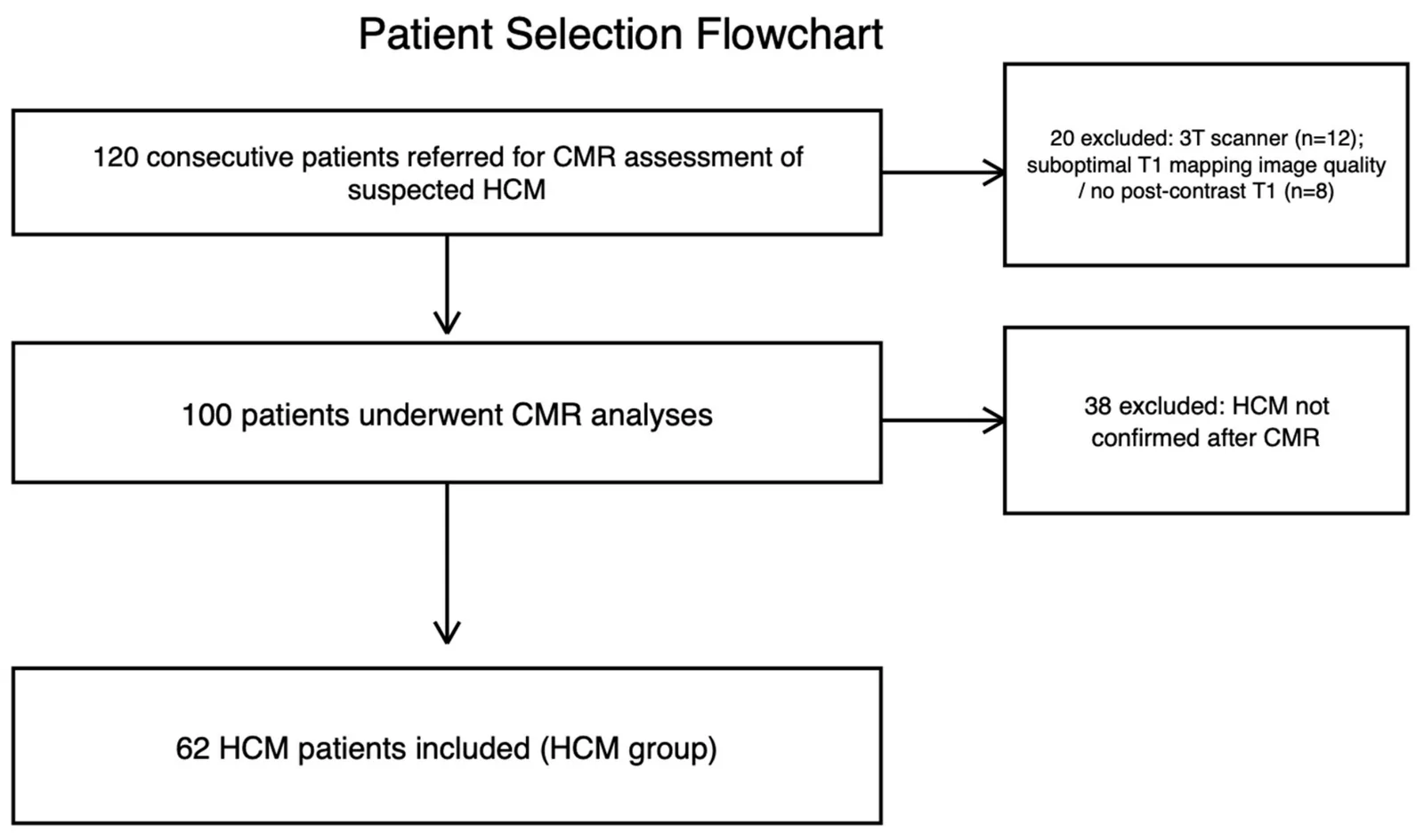
Patient selection flowchart.

A total of 120 consecutive patients were referred for assessment of suspected HCM. Of these, 58 were excluded either because HCM was not confirmed after comprehensive CMR evaluation or because pre-specified exclusion criteria were present. A total of 62 patients were enrolled in the HCM group (including apical HCM, limited/relative apical HCM, or other forms of HCM) (Figure 1).

The control group consisted of 38 age-matched patients, composed of 19 with normal CMR and 19 with HHD. The cohort consisted of 100 patients ranging between 21 and 90 years; 66% were male. The patient baseline characteristics and CMR parameters of the HCM vs. control group are summarized in Table 1.

There was no significant difference in mean LVEF between the HCM group (63 ± 6%) and the control group (64 ± 6%). LVMi was significantly higher in the HCM group (73 ± 2 g/m^2^) than in the control group (56 ± 1 g/m^2^) (Table 1).

Maximum end-diastolic wall thickness in the most hypertrophied segment was significantly higher in the HCM group (17 ± 5 mm) than in the control group (11± 3 mm) (Table 1). In the HCM group, the most hypertrophied LV segment distribution was 16 (26%) basal LV segments, 14 (23%) mid LV segments, and 32 (52%) apical LV segments. In the control group, the thickest LV segment distribution was 31 (82%) basal LV segments, seven (18%) mid LV segments, and 0 (0%) apical LV segments.

T2 mapping values were significantly higher in the HCM group than in the control group in the basal (53 ± 3 ms vs. 51 ± 3 ms, *p* = 0.029) and mid segments (52 ± 3 ms vs. 51 ± 3 ms, *p* = 0.034) (Table 1). Overall, late gadolinium enhancement (LGE) was present in 48 patients; all of them were in the HCM group, corresponding to 77% of the HCM patients.

### 3.2. T1 Mapping and ECV

Myocardial T1 values in the most hypertrophied segment were significantly higher in the HCM group than in the control group (1053 ± 48 ms vs. 1012 ± 38 ms, respectively, *p* < 0.001); the corresponding ECV fractions were also higher in the HCM group (29 ± 4% vs. 25 ± 3%, *p* < 0.001). Additionally, the mean native T1 values in mid and apical segments were significantly higher in the HCM group (Table 1).

### 3.3. LV Strain

Compared to the control group, global longitudinal strain (GLS) (−17.4 ± 2.9% vs. −13.5 ± 5.1%, *p* < 0.001), global circumferential strain (GCS) (−16.1 ± 5.9% vs. −20.8 ± 3.6%, *p* < 0.001), and global radial strain (GRS) (45.2 ± 14.1% vs. 32.1 ± 14.0%, *p* < 0.001) demonstrated lower function in HCM group (Table 1).

### 3.4. LV Function and Structure Correlation

Correlations between structural and functional CMR parameters in the hypertrophied myocardial segments of HCM and control groups are summarized in Table 2 and Table 3, respectively.

Significant correlation was found between structural and functional measurements in the HCM group. Regarding tissue characterization, significant correlation was found between T1 values at the hypertrophied segment and GLS (r = 0.27, *p* = 0.031), GCS (r = 0.34, *p* = 0.006), GRS (r = −0.29, *p* = 0.024) and LVEF (r = −0.31, *p* = 0.014), implying that higher T1 values were associated with worse GLS, GCS, GRS and LVEF. Conversely, no correlation was found between ECV at the hypertrophied segment and functional measurements. No correlation was found between most of the structural and functional measurements in the control group. In this group, a significant negative correlation was found between myocardial T1 values in the thicker segment and GLS (r = −0.37, *p* = 0.021) and LV mass index and GCS (r = −0.35, *p* = 0.032) and GRS (r = −0.33, *p* = 0.044).

In multivariable analysis, compared to the lowest tertile of native T1, the highest tertile of native T1 was independently associated with higher (i.e., worse function) GCS (adjusted mean difference = 3.98, 95%CI 0.28–7.68, *p* = 0.036) and GLS (adjusted mean difference = 3.36, 95%CI 0.30–6.42, *p* = 0.032).

## 4. Discussion

Our study demonstrated that T1 mapping values and ECV fractions were higher in the HCM group versus the control group, especially in the most hypertrophied myocardial segments. Regional circumferential and radial strain values in the most hypertrophied segment demonstrated significantly lower function in the HCM group when compared with the control group. In the HCM group, significant correlation was found between the function parameters (strain and LVEF) and T1 values at the most hypertrophied segment, maximum end-diastolic LV wall thickness, and indexed LV mass. Conversely, there was no significant correlation between most of the structural and functional parameters in the control group.

### 4.1. Relation Between LV Hypertrophy and Tissue Characterization

**Figure 2:**
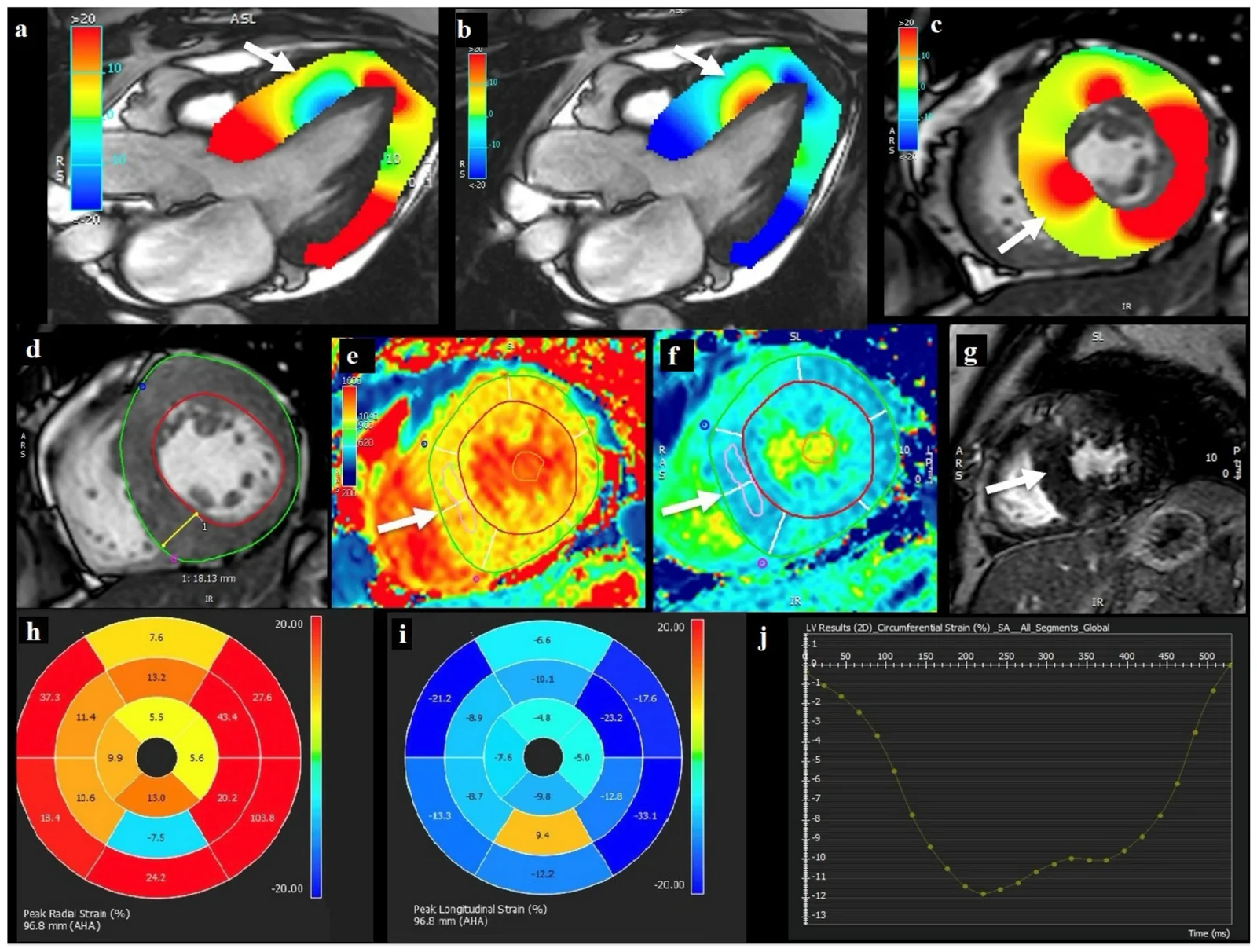
Native T1 tissue mapping and corresponding ECV maps and strain polar maps by feature tracking in a patient with asymmetric mid-ventricular septal hypertrophic cardiomyopathy. Representative color images of the contour of the radial (arrow in (**a**)), longitudinal (arrow in (**b**)), and circumferential (arrow in (**c**)) myocardial strain by feature tracking. Maximum end-diastolic wall thickness was 18 mm at the junction of the mid inferoseptal and inferior segments (**d**). Evaluation of native T1 (**e**) and ECV (**f**) values with region of interest (ROI) in the most hypertrophied mid inferoseptal segment away from the area of late gadolinium enhancement (**g**) showed an elevated T1 value of 1081 ms and ECV value of 32%. Representative polar maps of the global radial and longitudinal strains ((**h**) and (**i**), respectively), and peak systolic circumferential strain graph (**j**) revealed abnormal values in that segment (10.6%, −8.7%, and −12%, respectively).

**Figure 3:**
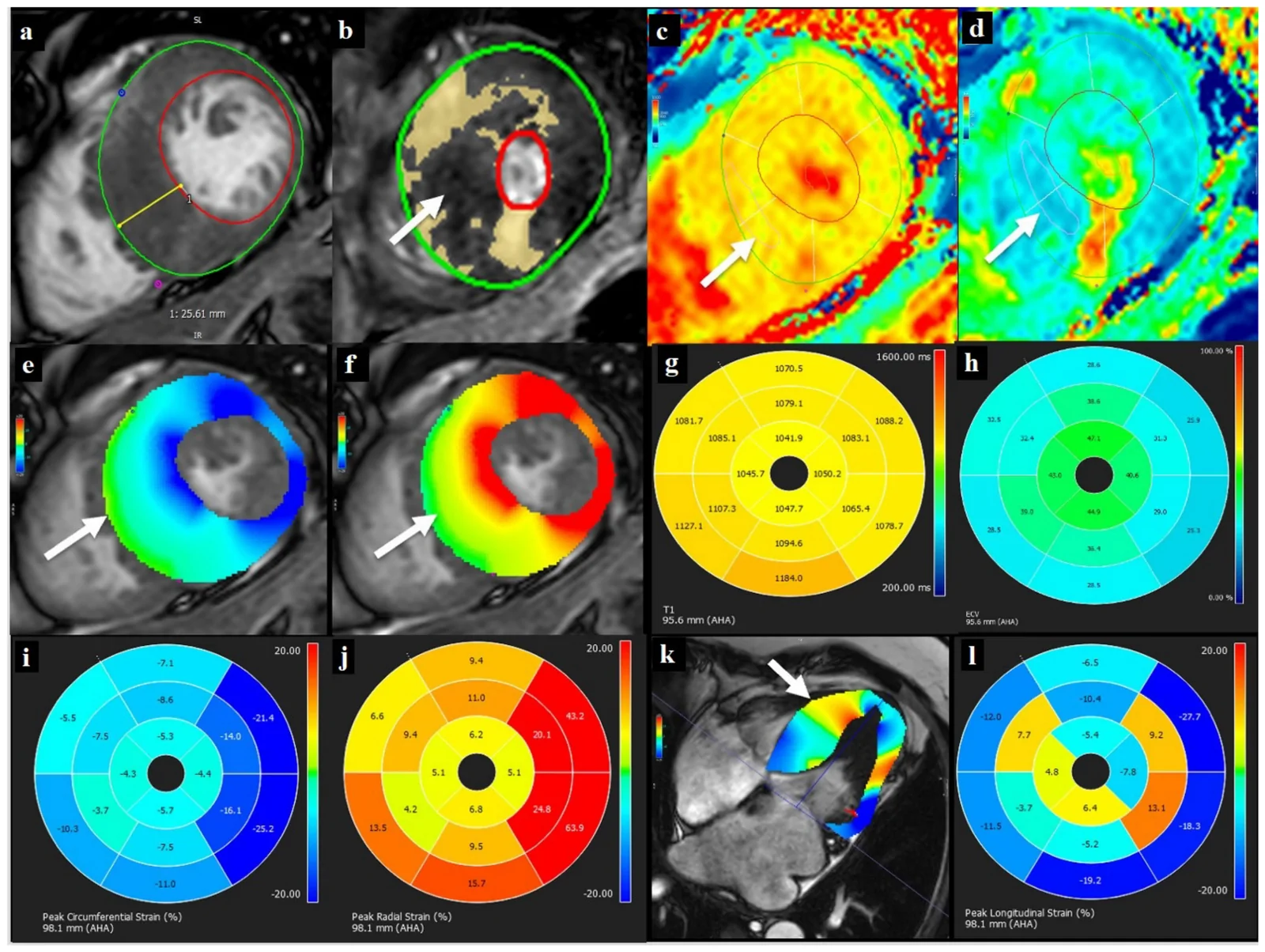
Native T1 tissue mapping and corresponding ECV maps and strain polar maps by feature tracking in a patient with significant asymmetric septal hypertrophic cardiomyopathy. Maximum end-diastolic wall thickness was 26 mm in the mid inferoseptal segment (**a**). Evaluation of native T1 and ECV values (arrows in (**c**,**d**)) with region of interest (ROI) in the most hypertrophied mid inferoseptal segment away from the area of late gadolinium enhancement (arrow in (**b**)) showed elevated values of 1088 ms and 32%, respectively, with corresponding polar maps (**g**,**h**). Representative color images and polar maps of the circumferential ((**e**) and (**i**) respectively), radial ((**f**) and (**j**) respectively), and longitudinal (arrow in (**k**) and (**l**)) myocardial strain by feature tracking revealed abnormal values in that segment (−3.7%, 4.2%, and −3.7% respectively).

Prior studies have shown that individuals with HCM exhibit higher global native T1 compared to healthy controls [31]. Additionally, there is a correlation between increased LV wall thickness and elevated native T1 time and ECV values. Our observations confirmed that native T1 and ECV values at the hypertrophied segment were significantly higher in HCM patients in comparison to native T1 and ECV values in the control group. Moreover, native T1 was significantly higher in the mid and apical segments in the HCM group. These findings reflect both the adverse myocardial and extracellular changes in HCM, affecting the hypertrophied segment as well as segments of non-hypertrophied myocardium, reinforcing the presence of fibrosis in both focal and diffuse forms (Figure 2 and Figure 3) [31,32].

### 4.2. Relation Between LV Structure and Function

Using FT-CMR, patients with HCM have worse longitudinal, radial, and circumferential strains compared with control subjects, while radial and longitudinal strains were predictive of clinical outcome [33,34]. Previous studies employing speckle tracking echocardiography (STE) strain assessment demonstrated similar differences in strain between HCM and HHD [28,35,36]. Similar to previous studies, we found a significant association between maximum end-diastolic wall thickness and LVMi and strain analysis. In this context, GLS demonstrated moderate correlation with the structural aspects, while in the control group no significant correlation was noted. A prior study demonstrated that in hypertensive patients, GLS is more abnormal in the presence of LVH and correlates with LV mass index [37], indicating that contractility worsens with disease progression. In HCM, GLS decline occurs even prior to phenotype development [38]. GRS was significantly impaired in HCM patients compared to control subjects. Impaired radial thickening in HCM may reflect disruption of the myofiber architecture by myocyte disarray, replacement fibrosis, and diffuse interstitial remodeling. At the segmental level, radial strain at the most hypertrophied segment was significantly lower in HCM compared to control individuals, highlighting that the site of greatest structural remodeling also bears the greatest mechanical impairment, consistent with the regional structure–function coupling.

While it may be postulated that more fibrosis (which occurs more in the thickened segments in HCM) is associated with worse function (strain), no prior study with a control group has specifically examined this. Our results revealed a significant correlation between T1 values in the hypertrophied segment and strain as well as with LVEF in HCM patients. In this group, increased T1 values demonstrated a correlation with worse strain, suggesting that the presence of fibrosis is associated with worse global function. Swoboda et al. [32] demonstrated associations between T1 mapping parameters and regional contractile function in patients with HCM, although it is not clear whether such relationships also exist in other conditions. By including a control group spanning a clinically relevant spectrum of LV remodeling, our study shows that the structure–function coupling observed is specific to the HCM myopathic substrate rather than a non-specific consequence of regional LV hypertrophy per se. In interpreting these structure–function relationships, quantitative LGE remains a widely used and clinically relevant marker in HCM studies, particularly for risk stratification and comparison across cohorts. However, LGE quantification is methodologically variable and potentially problematic in HCM. LGE simply reflects the relative differences in post-contrast T1 and ECV across the myocardium, and its detection depends on an arbitrary signal-intensity threshold to define abnormal enhancement. This is particularly limiting in HCM, where diffuse interstitial fibrosis may be present throughout the myocardium, including in segments that appear normal on LGE imaging, such that LGE may substantially underestimate the true fibrotic burden. In contrast, ECV provides a continuous quantitative measure of extracellular expansion that captures both focal replacement fibrosis, typically reflected by LGE, and diffuse interstitial fibrosis that may be present in regions without visually apparent LGE. Therefore, ECV incorporates much of the biological information represented by LGE while also extending beyond it. Furthermore, we demonstrated that structural changes at the most hypertrophied segment correlated with global strain parameters, suggesting that the degree of structural remodeling at the epicenter of HCM pathology reflects the underlying diffuse cardiomyopathic process that contributes to global contractile dysfunction. Conversely, there was no significant correlation between T1 values and function analysis in the control group in our study, except for a negative correlation with GLS. Diffuse fibrosis plays an important role in the pathophysiology of LV dysfunction, where reduced LV ejection fraction is correlated to the increase in ECV in patients with non-ischemic cardiomyopathy [23,39,40]. In our study, ECV in the hypertrophied segment was higher in the HCM group than in the control group. However, it did not show a correlation with LV strain and LVEF in either the HCM or control group. The absence of a significant correlation between ECV at the hypertrophied segment and LV strain or LVEF, in contrast to the significant correlations observed with native T1, is a noteworthy finding. Native T1 reflects a composite of myocardial tissue properties, encompassing both cellular and extracellular compartments, and is sensitive to a broad spectrum of myocardial changes including myocyte hypertrophy, disarray, edema, and diffuse fibrosis. In HCM, these processes coexist and collectively contribute to elevated native T1, which may explain its stronger coupling with regional mechanical dysfunction compared to ECV alone. ECV, by contrast, is specifically designed to quantify the extracellular compartment and is subject to additional sources of measurement variability (e.g., hematocrit fluctuations). Alternatively, the lack of a significant correlation between ECV and LV strain might be due to inadequate power in this study. Our findings reflect that in HCM patients, the LV segments with structural changes (higher T1 values and ECV fraction) have worse function (higher GLS). Although this observational, correlative study sought to gain mechanistic pathophysiological insight into the structural and functional changes in HCM, rather than to establish immediate clinical tools or establish causality, a deeper understanding of how regional structural remodeling translates into functional impairment in HCM may have important implications. Further studies are also needed to evaluate the effect of novel pharmacotherapy for HCM on myocardial structure and function, which can be well assessed by CMR.

### 4.3. Limitations

Our study has several limitations. First, this was a single-center, retrospective, cross-sectional study, which limits causal inference and precludes any prognostic analysis and assessment of temporal changes in LV structure and function and their evolving relationship over the disease course. Second, no formal a priori sample size calculation was performed. The cohort comprised all consecutive eligible patients at our institution during the study period. As a result, the study may have been underpowered to detect weaker correlations, particularly within morphological subgroups, and the observed marginal correlation coefficients should be interpreted accordingly. Due to the small sample size and limited statistical power, the results of the multivariable analysis should be considered exploratory and require confirmation in future larger studies. Furthermore, the limited sample size precludes adjustment for additional potential confounders beyond those included in our model. Larger multicentre prospective studies with multivariable analysis are warranted to validate these findings. Third, all CMR image analyses were performed by a single reader, and independent external validation of measurements was not performed. Fourth, the control group comprised healthy subjects and patients with hypertension; future studies using other control groups such as athletes who have physiological LV hypertrophy may yield novel insights. Additional studies are needed to confirm our hypothesis-generating findings, and to examine these structural and functional changes in relation to perfusion reserve and myocardial oxygenation [41], and their relative prognostic value. Longitudinal follow-up CMR studies would provide further insight into these dynamic processes.

## 5. Conclusions

Our study demonstrated significant correlations between global LV myocardial strain by CMR-FT and regional tissue characteristics as assessed by tissue mapping sequences at the site of the most hypertrophied myocardial segments in HCM. Future studies with larger sample sizes are warranted to corroborate these observations and determine the relative and potentially complementary prognostic value of structural and functional changes in HCM.

## Figures and Tables

**Table 1:** Baseline characteristics and CMR parameter values of HCM vs. control patients.

Group	Total Patients (*N* = 100)	HCM ^1^ (*N* = 62)	Control (*N* = 38)	*p*-Value
Age, y	56 ± 15	60 ± 15	52 ± 15	0.012
Male (%)	66 (66%)	69%	61%	-
LVEF ^2^, %	63 ± 6	63 ± 6	64 ± 6	0.48
LVEDVi ^3^, mL/m^2^	75 ± 18	74 ± 18	77 ± 18	0.37
LVESVi ^4^, mL/m^2^	29 ± 13	30 ± 15	29 ± 9	0.81
LV Mass, g	130 ± 48	142 ± 54	112 ± 30	0.002
LVMi ^5^, g/m^2^	66 ± 20	73 ± 23	56 ± 12	<0.001
Basal myocardial T1 value, ms	1022 ± 44	1028 ± 48	1012 ± 34	0.13
Mid myocardial T1 value, ms	1019 ± 46	1027 ± 47	1008 ± 43	0.050
Apical myocardial T1 value, ms	1020 ± 47	1031 ± 51	1002 ± 33	0.003
Basal myocardial T2 value, ms	52 ± 3	53 ± 3	51 ± 3	0.029
Mid myocardial T2 value, ms	52 ± 3	52 ± 3	51 ± 3	0.034
Apical myocardial T2 value, ms	52 ± 3	52 ± 3	52 ± 3	0.855
Basal ECV ^6^, %	26 ± 3	26 ± 3	25 ± 3	0.060
Mid ECV ^6^, %	26 ± 3	27 ± 3	25 ± 3	0.056
Apical ECV ^6^, %	27 ± 4	28 ± 4	26 ± 3	0.008
T1 hypertrophied segment, ms	1037 ± 48	1053 ± 48	1012 ± 38	<0.001
ECV ^6^ at hypertrophied segment, %	27 ± 4	29 ± 4	25 ± 3	<0.001
Maximum end-diastolic wall thickness, mm	14 ± 4	17 ± 5	11 ± 3	<0.001
GLS ^7^, %	−15.0 ± 4.8	−13.5 ± 5.1	−17.4 ± 2.9	<0.001
GCS ^8^, %	−17.9 ± 5.6	−16.1 ± 5.9	−20.8 ± 3.6	<0.001
GRS ^9^, %	37.1 ± 15.3	32.1 ± 14.0	45.2 ± 14.1	<0.001
Basal LS ^10^, %	−14.6 ± 5.4	−13.2 ± 5.7	−16.9 ± 3.9	<0.001
Mid LS ^10^, %	−15.0 ± 5.1	−13.3 ± 5.2	−17.9 ± 3.4	<0.001
Apical LS ^10^, %	−15.5 ± 5.3	−14.4 ± 5.8	−17.3 ± 3.8	0.009
Basal CS ^11^, %	−17.2 ± 6.5	−16.2 ± 7.7	−18.8 ± 3.5	0.058
Mid CS ^11^, %	−18.0 ± 5.8	−16.3 ± 6.0	−20.9 ± 3.9	<0.001
Apical CS ^11^, %	−20.0 ± 8.0	−16.8 ± 8.0	−25.2 ± 4.3	<0.001
Basal RS ^12^, %	39.8 ± 16.9	37.0 ± 18.2	44.4 ± 13.5	0.009
Mid RS ^12^, %	36.1 ± 15.9	31.7 ± 14.8	43.3 ± 15.1	<0.001
Apical RS ^12^, %	45.2 ± 25.7	35.4 ± 23.4	61.1 ± 21.1	<0.001
LS ^10^ at hypertrophied segment, %	−9.4 ± 15.2	−9.0 ± 12.8	−9.9 ± 18.7	0.065
CS ^11^ at hypertrophied segment, %	−12.9 ± 12.0	−12.2 ± 9.4	−14.0 ± 15.5	0.037
RS ^12^ at hypertrophied segment, %	29.7 ± 24.4	24.1 ± 19.8	39.1 ± 28.4	0.002

Data are expressed as mean ± SD ^13^ or frequencies and percentages. For parameters reported “at hypertrophied segment”, this refers to the most hypertrophied LV segment, defined as the segment with the greatest end-diastolic wall thickness on short-axis cine images. ^1^ hypertrophic cardiomyopathy; ^2^ left ventricular ejection fraction; ^3^ indexed left ventricular end diastolic volume; ^4^ indexed left ventricular end systolic volume; ^5^ indexed left ventricular mass; ^6^ extracellular volume; ^7^ global longitudinal strain; ^8^ global circumferential strain; ^9^ global radial strain; ^10^ longitudinal strain; ^11^ circumferential strain; ^12^ radial strain; ^13^ standard deviation.

**Table 2:** Spearman correlation coefficients between cardiac MRI structural and functional measurements in the HCM group.

			Function		
Structure		**GLS** ^2^	**GCS** ^3^	**GRS** ^4^	**LVEF** ^5^
	Myocardial T1 value at hypertrophied segment (*n* = 62)	0.27 (*p* = 0.031)	0.34 (*p* = 0.006)	−0.29 (*p* = 0.024)	−0.31 (*p* = 0.014)
	ECV ^1^ at hypertrophied segment (*n* = 61)	0.03 (*p* = 0.82)	0.08 (*p* = 0.56)	−0.04 (*p* = 0.75)	−0.12 (*p* = 0.36)
	Maximum end diastolic wall thickness (*n* = 62)	0.41 (*p* = 0.001)	0.43 (*p* < 0.001)	−0.34 (*p* = 0.006)	−0.35 (*p* = 0.006)
	LV mass index (*n* = 62)	0.49 (*p* < 0.001)	0.41 (*p* = 0.001)	−0.39 (*p* = 0.002)	−0.53 (*p* < 0.001)

^1^ extracellular volume; ^2^ global longitudinal strain; ^3^ global circumferential strain; ^4^ global radial strain; ^5^ left ventricular ejection fraction.

**Table 3:** Spearman correlation coefficients between cardiac MRI structural and functional measurements in the control group.

			Function		
Structure		**GLS** ^2^	**GCS** ^3^	**GRS** ^4^	**LVEF** ^5^
	Myocardial T1 value at thicker segment (*n* = 38)	−0.37 (*p* = 0.021)	−0.17 (*p* = 0.32)	0.13 (*p* = 0.43)	0.15 (*p* = 0.37)
	ECV ^1^ at thicker segment *(n* = 38)	−0.18 (*p* = 0.28)	−0.08 (*p* = 0.66)	0.09 (*p* = 0.59)	0.02 (*p* = 0.91)
	Maximum end diastolic wall thickness (*n* = 38)	−0.26 (*p* = 0.12)	−0.14 (*p* = 0.40)	0.23 (*p* = 0.17)	0.11 (*p* = 0.50)
	LV mass index (*n* = 38)	0.29 (*p* = 0.079)	−0.35 (*p* = 0.032)	−0.33 (*p* = 0.044)	−0.31 (*p* = 0.060)

^1^ extracellular volume; ^2^ global longitudinal strain; ^3^ global circumferential strain; ^4^ global radial strain; ^5^ left ventricular ejection fraction.

## Data Availability

The datasets used and/or analyzed during the current study are available from the corresponding authors on reasonable request.

## Abbreviations

The following abbreviations are used in this manuscript:

bSSFP	balanced steady-state free precession
CMR	cardiovascular magnetic resonance
CMR-FT	cardiovascular magnetic resonance-feature tracking
ECG	electrocardiographic
ECV	extracellular volume
GCS	Global circumferential strain
GLS	Global longitudinal strain
GRS	Global radial strain
HCM	hypertrophic cardiomyopathy
HHD	hypertensive heart disease
LGE	late gadolinium enhancement
LVWT	left ventricular wall thickness
LVEDVi	left ventricular end diastolic volume index
LVESVi	left ventricular end systolic volume index
LVSV	left ventricular stroke volume
LVEF	left ventricular ejection fraction
LVMi	left ventricular mass index
